# Bazedoxifene enhances paclitaxel efficacy to suppress glioblastoma via altering Hippo/YAP pathway

**DOI:** 10.7150/jca.38350

**Published:** 2020-01-01

**Authors:** Weiwei Fu, Peng Zhao, Hong Li, Haiyang Fu, Xuejun Liu, Yingchao Liu, Jie Wu, Weiwei Fu

**Affiliations:** 1 Department of Pathology, The Affiliated Hospital of Qingdao University, Qingdao, Shandong 266003, P. R. China;; 2 Department of Radiology, The Affiliated Hospital of Qingdao University, Qingdao, Shandong 266003, P. R. China;; 3 Department of Neurosurgery, Provincial Hospital Affiliated to Shandong University, Jinan, Shandong 250006, P. R. China.

**Keywords:** Bazedoxifene, Paclitaxel, Glioblastoma, YAP

## Abstract

Glioblastoma multiform (GBM) is an aggressive type of brain tumor originated from astrocytes. Owing to the limited therapeutic options, intensive efforts are still being made to find novel treatments for GBM. In this study, we first identified that bazedoxifene bore the ability to reduce cell survival and cell invasion of glioblastoma cells. Furthermore, our results also revealed that bazedoxifene combining with paclitaxel had better efficacy to suppress glioblastoma progression by promoting apoptosis and reducing EMT. Combination of bazedoxifene and paclitaxel also accelerated YAP phosphorylation and inactivation. Importantly, preclinical animal model also verified our in vitro findings. Together, our data not only define the underlying mechanism responsible for action of bazedoxifene on glioblastoma cells but also build strong rational to develop bazedoxifene for the treatment of GBM patients.

## Introduction

Glioblastoma multiform (GBM) is the most common brain tumor and it occurs in adults with approximate incidence at 3.2/100 000 12, 18. The mainstay treatments for glioblastoma are surgery, radiotherapy and chemotherapy 15. Although these current therapies show promising effectiveness, GBM patients still have poor survival rate with a median survival of only 15 months 16. Therefore, intensive studies are focused on finding novel targeted therapies for this disease.

Bazedoxifene is a third-generation selective estrogen receptor modulator (SERM) and has approved by the FDA to treat postmenopausal osteoporosis 10. In addition, bazedoxifene is also being tested for possible treatment of breast cancer, medulloblastoma and pancreatic cancer 3, 6, 19, 23. These studies have demonstrated that bazedoxifene acts as GP130 inhibitor by competing with IL-6 or IL-11 for the interaction of GP130, leading to the deactivation of IL-6/GP130 signaling and delayed cancer progression. Bazedoxifene treatment in medulloblastoma cells suppressed cell growth and impaired glycolysis 3. In triple negative breast cancer cells, bazedoxifene also showed an anti-cancer activity by suppressing cell proliferation, cell invasion and tumor growth 19. However, the anti-cancer activity of bazedoxifene in glioblastoma and whether it can enhance paclitaxel efficacy to suppress glioblastoma cell growth haven't yet been investigated.

Numerous studies have demonstrated that Hippo signaling is involved in cancer development including glioblastoma 27. YAP is one of essential components in Hippo signaling cascade. MST1/2 (mammalian sterile 20-like kinases 1/2) complexes with Sav1 (salvador family WW domain-containing protein 1) to activate LATS1/2 (large tumor suppressor kinases 1/2), leading to the phosphorylation and degradation of YAP 26, 28. While the dephosphorylated YAP works together with many transcription factors to control gene expression such as CTGF and Cyr61 2. Amplification of YAP has been reported in several human cancers 25. YAP overexpression could promote epithelial- mesenchymal transition (EMT) in MCF10A cells and promoted tumor growth of liver cancer 14, 24. All these findings have established the important role of the Hippo pathway in cancer development. Although the significance of YAP has been documented in glioblastoma, small molecules targeting YAP are still intensively being tested.

In this study, we found that bazedoxifene alone or in combination with paclitaxel could suppress cell survival and cell invasion of glioblastoma cells. In vivo mouse model also confirmed that combinational treatment using bazedoxifene and paclitaxel further suppressed glioblastoma tumor growth compared with single treatment. Our data provided rational to develop bazedoxifene as another therapeutic option for glioblastoma, either alone or in combination with other drugs.

## Materials and Methods

### Cell culture

LN229, D456, OMRP, LN18, 1016, U251MG and astrocyte cell lines were purchased from ATCC. Cells were cultured in Dulbecco's modified Eagle's medium (DMEM) (Corning, 10-013-CV) supplemented with 10% Fetal Bovine Serum (Gibco, 10437028) and 1% Antibiotic-Antimycotic Solution (Corning, 30-004-CI). Bazedoxifene acetate and paclitaxel were purchased from Acesys Pharmatech (USA) and dissolved in DMSO at the concentration of 20 mM and 5 mg/ml respectively. ERα, Cyclin D, Bcl-2, p-p70S6K, Vimentin, E-cadherin, p-YAP (S127), CTGF, Cyr61, sail, MMP9 and Actin antibodies were purchased from Cell Signaling Technology (Beverly, Massachusetts, USA).

### Cell viability assay

Cell viability was measured using 3-(4, 5-dimethylthiazolyl)-2 and 5-diphenyltetrazolium bromide (MTT) assay. Glioblastoma cells were seeded in 96-well plates at a density of 5000 cells per well. 24 hours later, cells were treated by bazedoxifene or paclitaxel or combination for another 24 hours. Then, 25 μl of MTT (Sigma, St Louis, Missouri, USA) was added to each well and incubated for 2 hours. Relative cell number was measured by the absorbance at 570 nm.

### Western blotting

Cell were lysed by RIPA lysis buffer. Equal amount of protein (20 µg) was subjected to 8%-12% SDS-PAGE gel separation and transferred to a polyvinylidene difluoride membrane. After being blocked by 10% BSA, the membranes were incubated with specific primary antibodies at 4°C overnight. Then the membranes were washed by Tris-buffered saline with 0.1% Tween-20 and incubated with corresponding secondary antibody at room temperature for 1 h. Blots were detected using the ChemiDOC XRS + system (Bio-Rad Laboratories, Hercules, California, USA).

### Colony formation assay

Glioblastoma cells were seeded into 6-cm culture plate at the density of 1000. Cells were treated by either bazedoxifene or paclitaxel or combination. 2 weeks later, colonies were fixed with cold methanol and stained with 0.1% crystal violet.

### 3D invasion assay

Precoat 8-well glass chamber slides with 40 μl of matrigel and allow them to solidify in the cell culture incubator. Seed 1 × 10^5^ glioblastoma cells into each well with medium containing 5% matrigel and 10 ng/ml EGF. Medium was replenished every 4 days. 2 weeks later, representative images were taken under 200× microscope. Acini-like structures were counted by randomly choosing six fields.

### Orthotopic GBM mouse model

1x10^6^ cells luciferase- cells were directly injected into the brain of six- to 6-week old nude mice. When tumor size reached to appropriate size, mice were divided into four groups and received the following treatments: 1) vehicle; 2) bazedoxifene (5 mg/kg); 3) paclitaxel (10 mg/kg); 4) bazedoxifene plus paclitaxel. Drugs were administered every other day. Tumor size was measured by IVIS. Four weeks later, mice were sacrificed for multiple experiments.

### Statistical analysis

Statistical analysis was carried out using GraphPad Prism 6.0 software (GraphPad Software, San Diego, California, USA). Data was shown as mean ± SE. Differences were analyzed using Student t-test and significance was set at P value< 0.05. Each experiment was repeated at least three times.

## Results

### Bazedoxifene exhibited anti-cancer activity on glioblastoma cells

**Figure 1:**
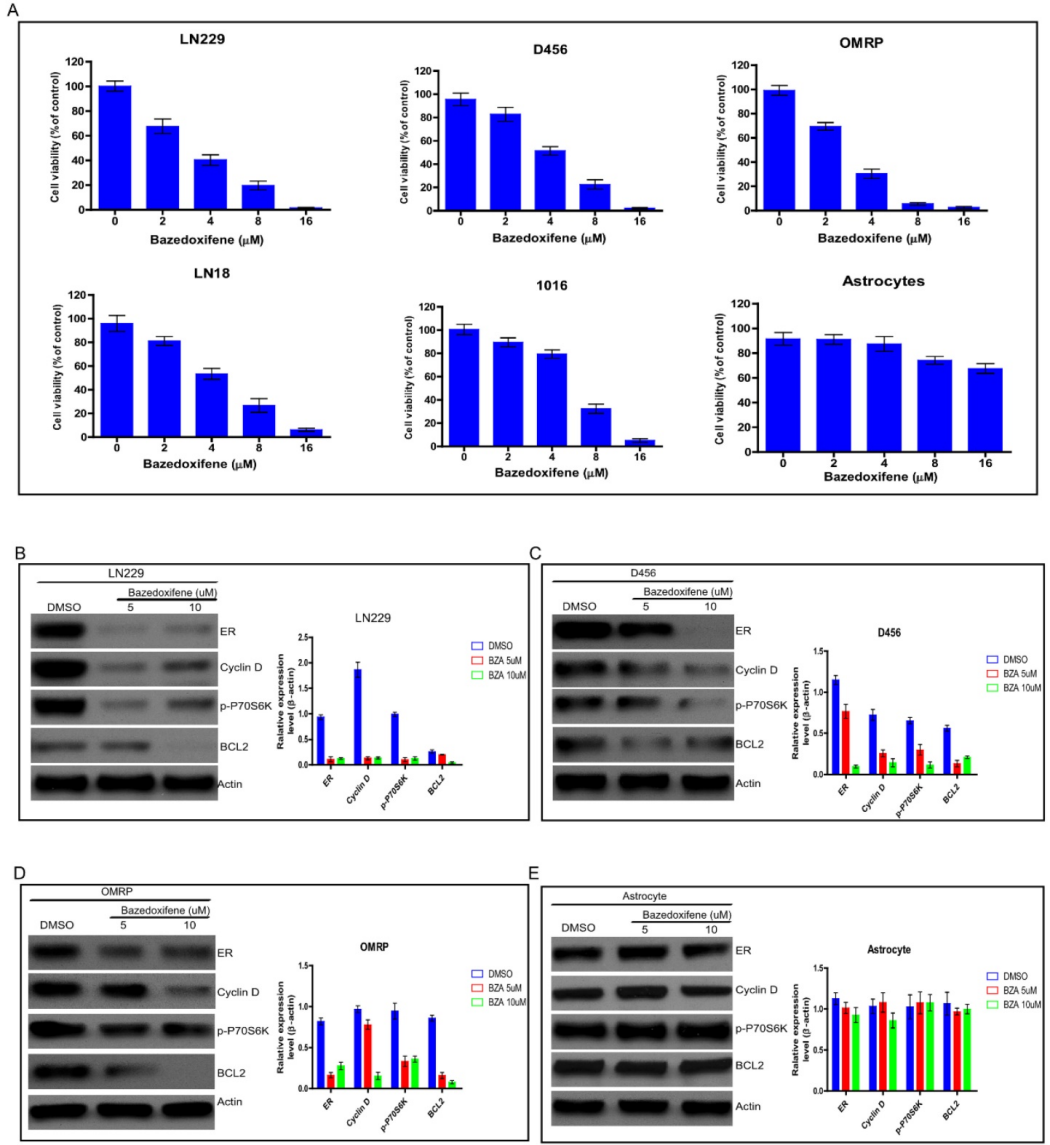
Bazedoxifene exhibited anti-cancer activity on glioblastoma cells. **(A)**. Bazedoxifene dose dependently suppressed cell viability of LN229, D456, OMRP, LN18 and 1016 cells but not normal astrocytes. **(B-D)**. Bazedoxifene treatment reduced the expression levels of ERα, Cyclin D, Bcl2 and suppressed mTOR activity in LN299 cells (B). D456 cells (C) and OMRP cells (D). ACTIN served as loading control. **(E)**. Bazedoxifene had little effect on the expression levels of ERα, Cyclin D, Bcl2 in astrocytes. ACTIN was internal control. *p<0.1; **p<0.01; ***p<0.001.

Bazedoxifene has been approved to suppress bone tumor, breast cancer and pancreatic cancer in vitro and in vivo. To test whether bazedoxifene exerted anti-cancer activity on glioblastoma cells, we treated several glioblastoma cell lines (LN229, D456, OMRP, LN18 and 1016) with bazedoxifene for 24 hours. Data showed that bazedoxifene decreased cell viability of these glioblastoma cells in a dose dependent manner (**Figure 1A**). As control, normal astrocytes failed to respond to bazedoxifene (**Figure 1A**), indicating bazedoxifene bore an anti-cancer activity on glioblastoma cells without affecting normal cells. At molecular level, bazedoxifene treatment led to downregulation of ERα, Cyclin D, Bcl-2 and inactivation of mTOR signaling monitored by p-P70S6K (**Figure B-D**) in LN229, D456 and OMRP cells. Consistently, no apparent alteration of ERα, Cyclin D, Bcl-2 and mTOR was observed in astrocytes in the presence of bazedoxifene (**Figure 1E**).

### Bazedoxifene combined with paclitaxel synergistically suppressed cell growth of glioblastoma cells

**Figure 2:**
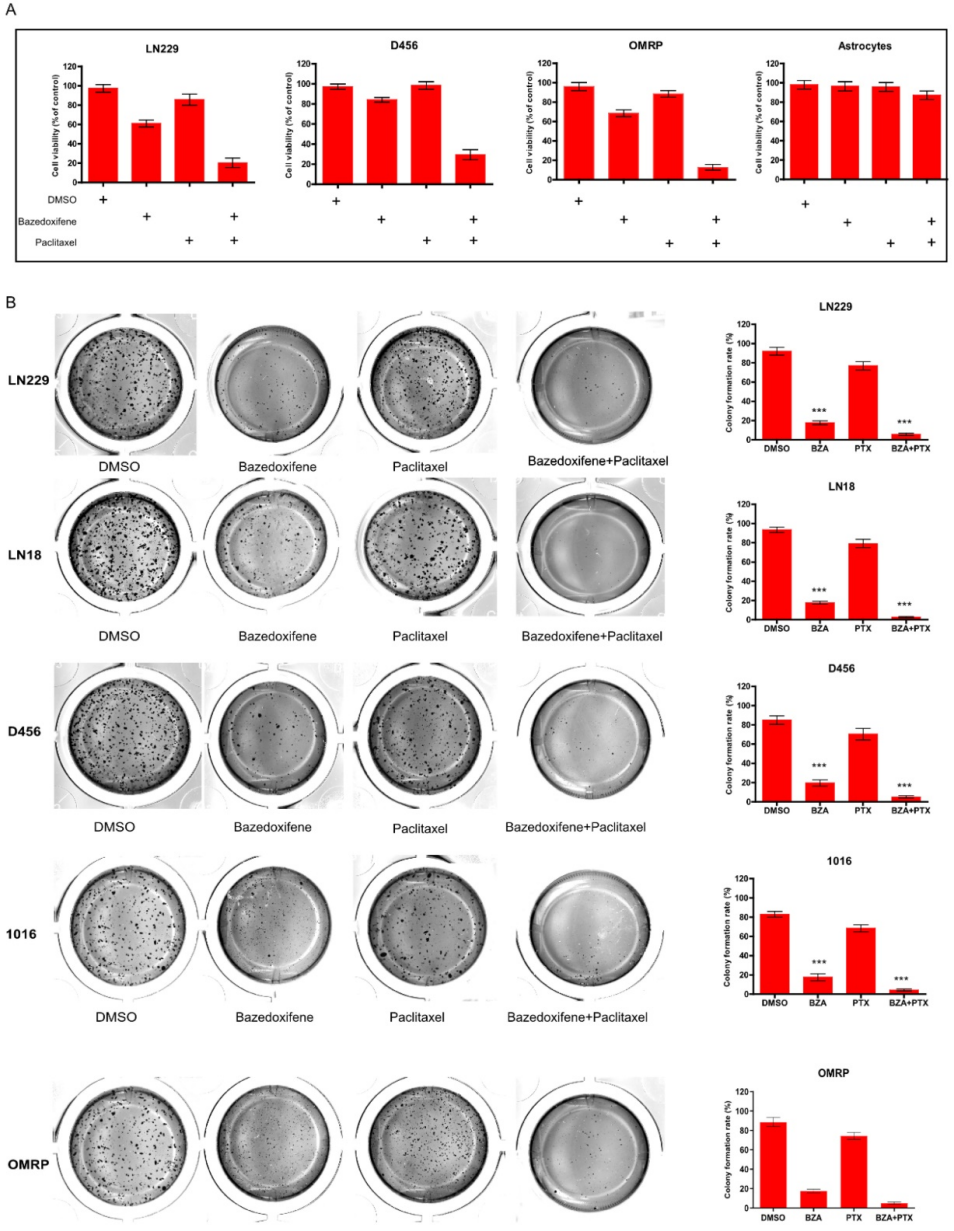
Bazedoxifene combined with paclitaxel synergistically suppressed cell growth of glioblastoma cells. **(A)**. Combination of bazedoxifene (5 µM) with paclitaxel (1 µg/ml) suppressed cell viability of LN229, D456, OMRP cells but not astrocytes. **(B)**. Colony formation assay revealed that bazedoxifene (5 µM) combined with paclitaxel (1 µg/ml) dramatically reduced cell growth of LN229, D456, OMRP, 1016 and LN18 cells. Left, representative images of colonies with indicated treatments. Right, statistical analyses. *p<0.1; **p<0.01; ***p<0.001.

Paclitaxel is one of potential chemotherapy drugs used for the treatment of glioblastoma patients owing to its anti- microtubule property. Here, we sought to investigate whether combination of paclitaxel with bazedoxifene had better efficacy to suppress glioblastoma cells. Expectedly, bazedoxifene combined with paclitaxel had better ability to decrease cell survival of glioblastoma cells compared to single treated group, assayed by MTT in LN229, D456 and OMRP cells (**Figure 2A**). As control, astrocytes had no response to these two drugs (**Figure 2A**). Accordantly, colony formation assay also confirmed that bazedoxifene combined with paclitaxel evidently reduced the number of colonies in LN229, D456, OMRP, LN18 and 1016 cells (**Figure 2B**).

### Bazedoxifene combined with paclitaxel triggered a stronger cell apoptosis

**Figure 3:**
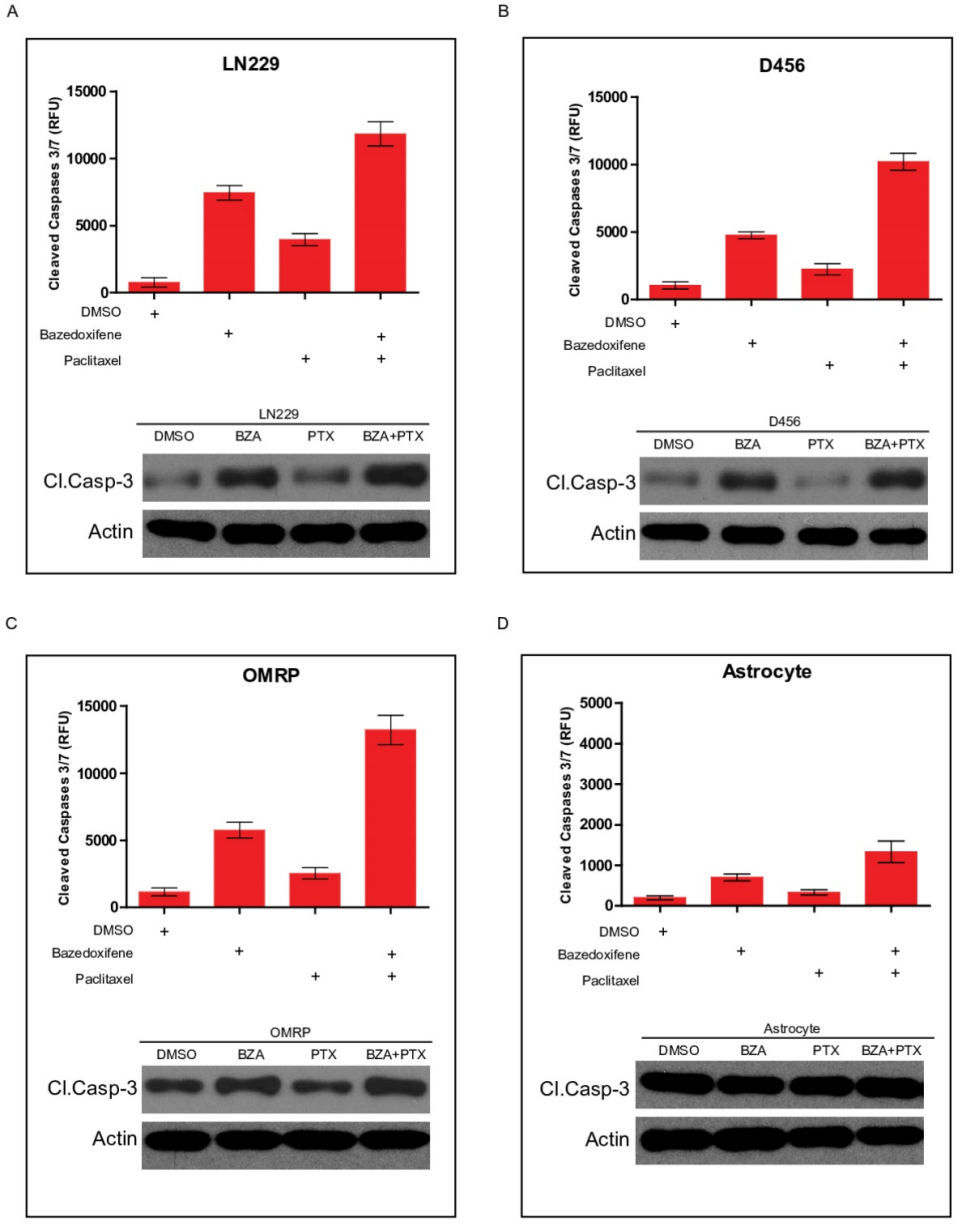
Bazedoxifene combined with paclitaxel triggered a stronger cell apoptosis. Bazedoxifene: 5 µM; Paclitaxel: 1 µg/ml. **(A-C)**. Western blotting results showed that combination treatment using bazedoxifene (BZA) and paclitaxel (PTX) had stronger ability to promote cleaved caspase-3 production in LN229 **(A)**, D456 **(B)** and OMRP cells **(C)**. Top, statistical analyses of cleaved caspase-3 blots. Bottom, western blotting detection of cleaved caspase 3 and ACTIN. ACTIN was internal control. **(D)**.Neither bazedoxifene nor paclitaxel had effect on the expression levels of cleaved caspase 3 in astrocytes. ACTIN was used as loading control. *p<0.1; **p<0.01; ***p<0.001

Apoptosis pathway is well linked to cell growth. We next sought to test whether cell apoptosis was involved in bazedoxifen/paclitaxel mediated cell growth suppression of glioblastoma cells. Cleaved caspase-3 serves as one marker of cell apoptosis. As expected, bazedoxifene alone promoted cleaved caspase-3 production in LN229 cells. However, the combination of bazedoxifene with paclitaxel had stronger ability to elevate cleaved caspase-3 levels (**Figure 3A**), suggesting cell apoptosis was involved in bazedoxifen/paclitaxel mediated cell growth suppression of glioblastoma cells. Similar results were obtained when we replaced LN229 cells with D456 (**Figure 3B**) or OMRP cells (**Figure 3C**). As control, neither bazedoxifene nor paclitaxel had effect on cleaved caspase-3 levels in normal astrocyte cells (**Figure 3D**).

### Bazedoxifene combined with paclitaxel further inhibited cell invasion of glioblastoma cells

**Figure 4:**
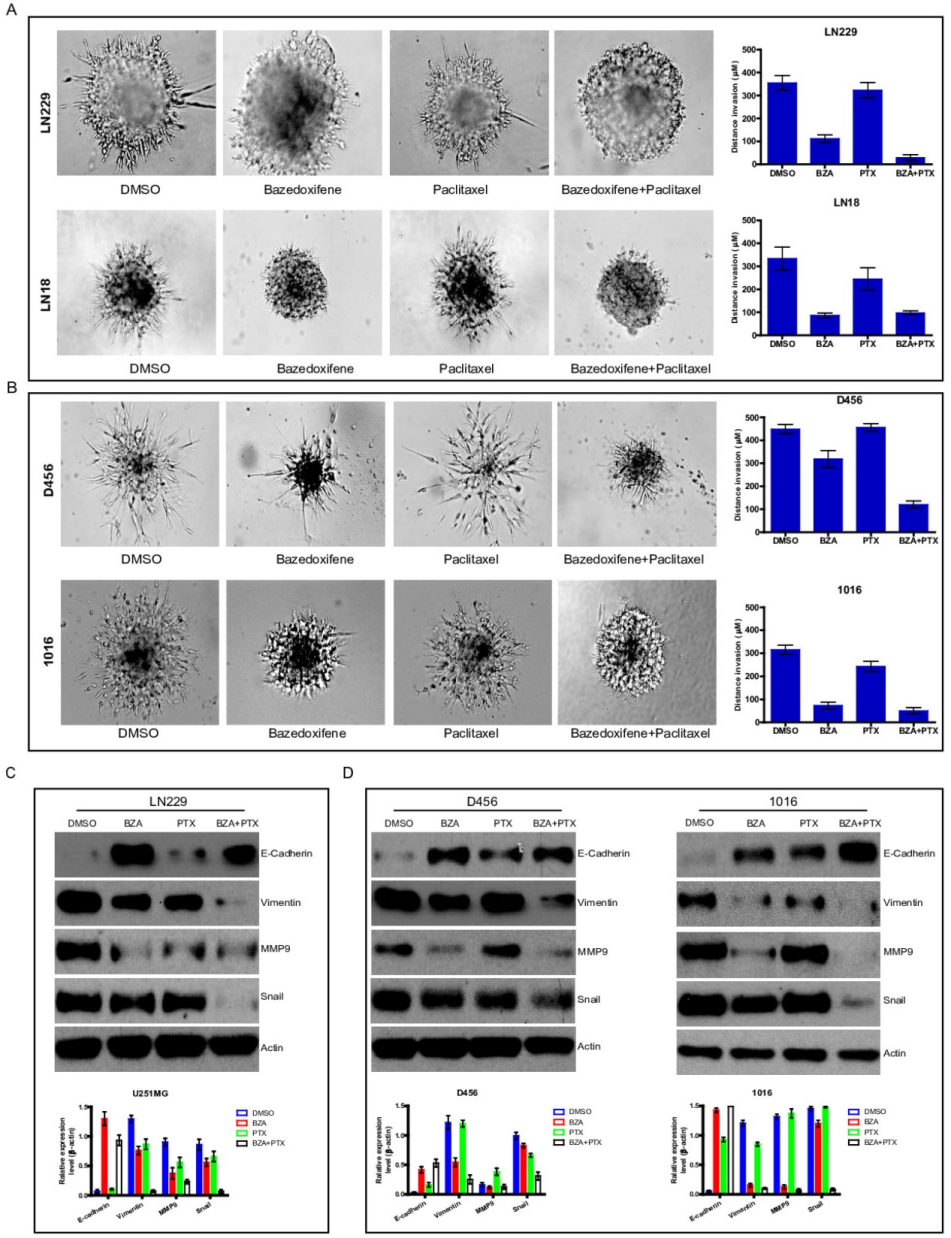
Bazedoxifene combined with paclitaxel further inhibited cell invasion of glioblastoma cells. Bazedoxifene: 5 µM; Paclitaxel: 1 µg/ml. **(A)**. 3-D invasion indicated that bazedoxifene combined with paclitaxel further inhibited cell invasion of LN229 and LN18 cells. Left, representative images. Right, statistical analyses. **(B)**. 3-D invasion showed that bazedoxifene combined with paclitaxel further reduced cell invasion of D456 and 1016 cells. Left, representative images. Right, statistical analyses. **(C)**. Western blotting analyses demonstrated that combination of bazedoxifene with paclitaxel decreased expression levels of vimentin, MMP9 and snail but increased expression levels of E-cadherin in LN299 cells. **(D)** Real time qPCR analyses demonstrated that combination of bazedoxifene with paclitaxel decreased expression levels of vimentin, MMP9 and snail but increased expression levels of E-cadherin in D456 and 1016 cells. *p<0.1; **p<0.01; ***p<0.001

Cell invasion is one of essential hallmarks for cancer development. Next, we explored whether bazedoxifene combined with paclitaxel had any effect on cell invasion of glioblastoma cells. To end this, we applied 3-D invasion assay in glioblastoma cells as indicated treatments. Result showed that less acini-like structures were observed in bazedoxifene-treated and paclitaxel treated LN299 cells (**Figure 4A**). Importantly, combination of bazedoxifene with paclitaxel significantly reduced acini-like structures in LN299 cells (**Figure 4A, top**). We observed similar phenomenon in LN18, D456 and 1016 cells (**Figure 4A, bottom & Figure 4B**). Given the fact epithelial mesenchymal transition (EMT) plays essential role in the process of cell invasion, we tested several EMT markers with or without bazedoxifene/paclitaxel treatment. **Figure 4C-D** displayed that combinational treatment using bazedoxifene and paclitaxel caused considerable upregulation of E-cadherin and downregulation of vimentin, MMP9 and snail in LN229, D456 and 1016 cells, which was consistent to the phenotype we observed.

### Bazedoxifene combined with paclitaxel had stronger ability to suppress YAP signaling

**Figure 5:**
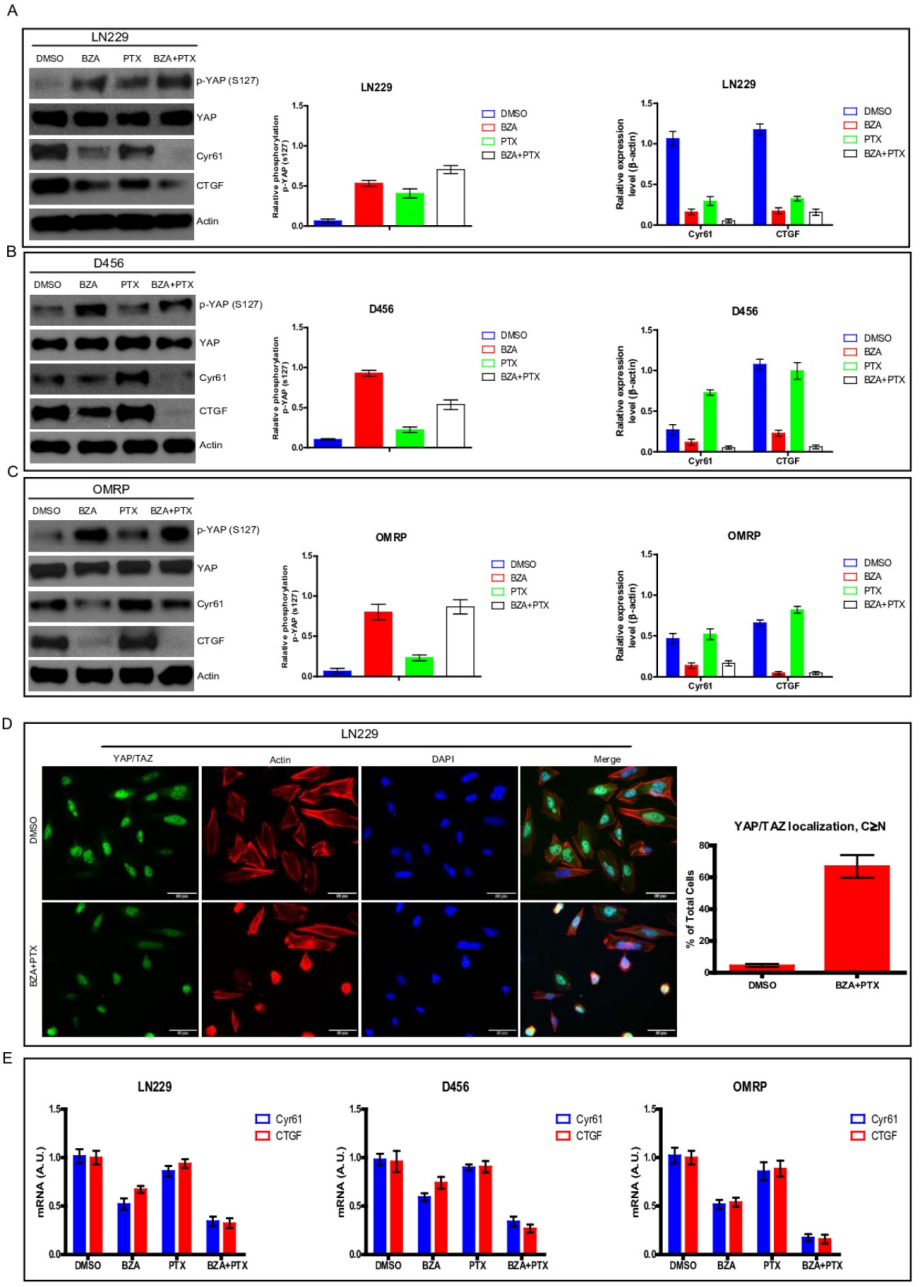
Bazedoxifene combined with paclitaxel had stronger ability to suppress YAP signaling. Bazedoxifene: 5 µM; Paclitaxel: 1 µg/ml.** (A-C)**.The phosphorylation levels of YAP (S127) were increased while the expression levels of CTGF and Cyr61 were decreased upon combinational treatment of bazedoxifene (BZA) and paclitaxel (PTX) in LN299 (A), D456 (B) and OMRP cells (C). **(D)**. immunofluorescence staining showed that bazedoxifene combined with paclitaxel led to accumulation of YAP in cytosol. **(E)**. Real time qPCR showed that the mRNA levels of CTGF and Cyr61 were reduced upon combinational treatment using bazedoxifene and paclitaxel. *p<0.1; **p<0.01; ***p<0.001

Hippo signaling pathway is involved in numerous biological processes including cell proliferation, organ development 7, 8. Hippo pathway is also tightly associated with cancer carcinogenesis and development 25. To test whether alteration of Hippo pathway is responsible for the suppressing effect of bazedoxifene and paclitaxel on glioblastoma cells, we used YAP phosphorylation and its downstream targets (CTGF and Cyr61) as indicators to monitor Hippo pathway. Results showed that bazedoxifene or paclitaxel alone could increase the phosphorylation levels of YAP and decrease the expression levels of CTGF and Cyr61 (**Figure 5A-C**) in LN229, D456 and OMRP cells. However, combination treatment using bazedoxifene and paclitaxel could further reduce the expression levels of CTGF and Cyr61 (**Figure 5A-C**). To confirm this finding, we also performed immunofluorescence staining to monitor YAP location. **Figure 5D** revealed that bazedoxifene plus paclitaxel treatment significantly boosted the translocation of YAP from nucleus to cytosol. Meanwhile, real-time qPCR also verified that bazedoxifene combined with paclitaxel had better capacity to reduce the mRNA levels of CTGF and Cyr61 compared to single treatment (**Figure 5E**). Together, all these data suggest that activation of Hippo pathway is at least one of mechanisms responsible for bazedoxifene-mediated cell growth suppression in glioblastoma cells.

### Bazedoxifene combined with paclitaxel further suppressed glioblastoma tumor growth

**Figure 6:**
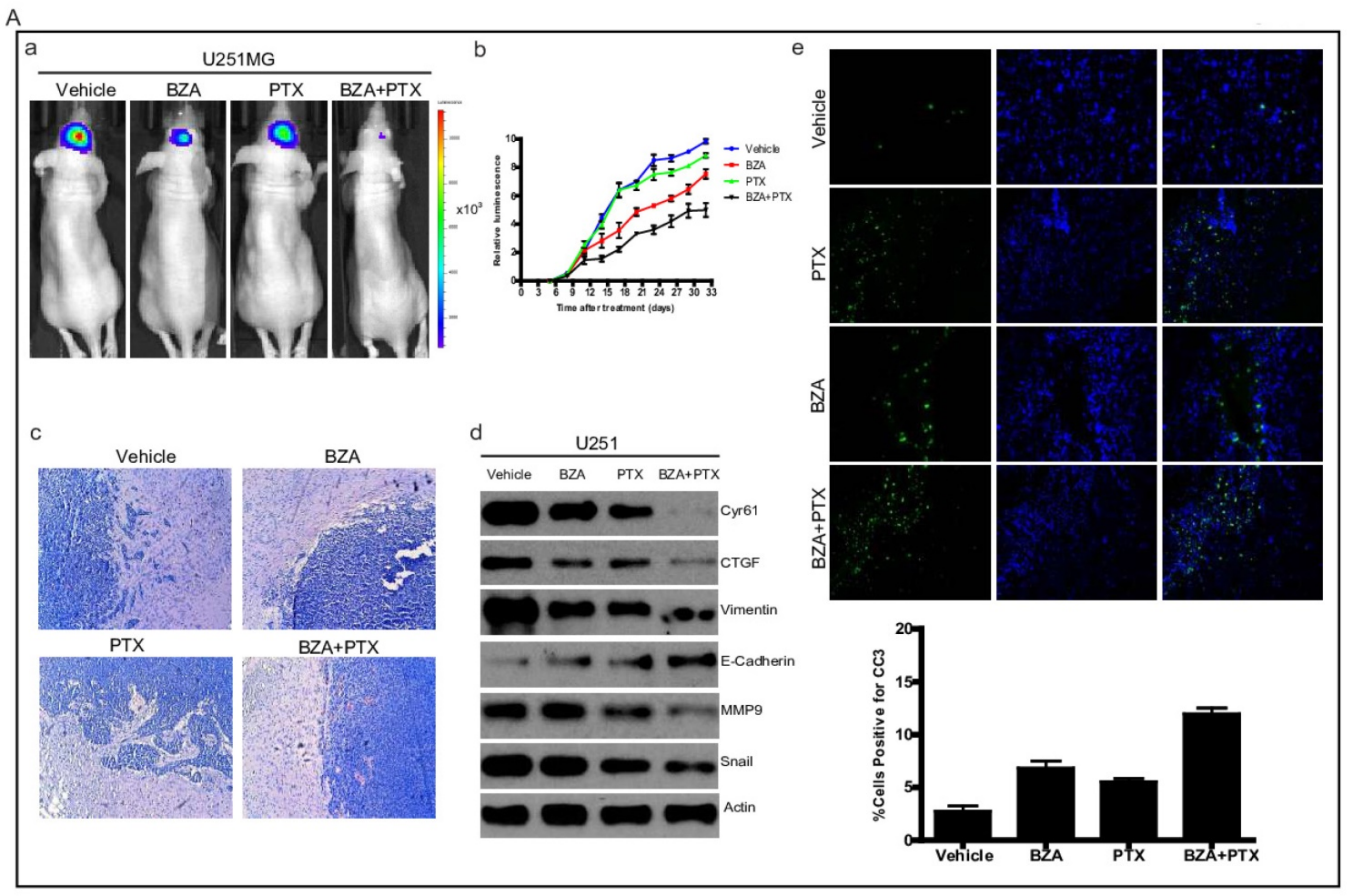

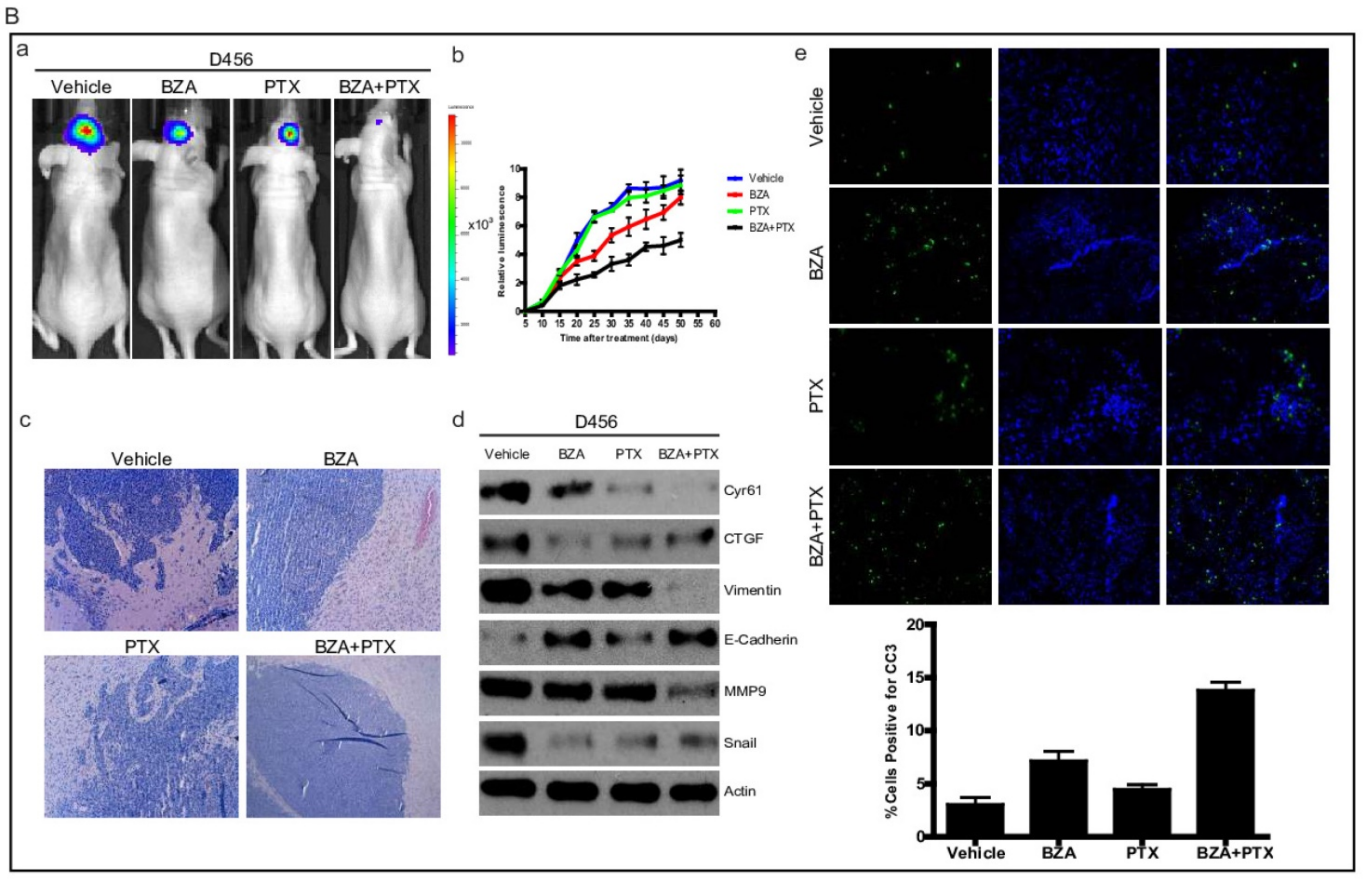

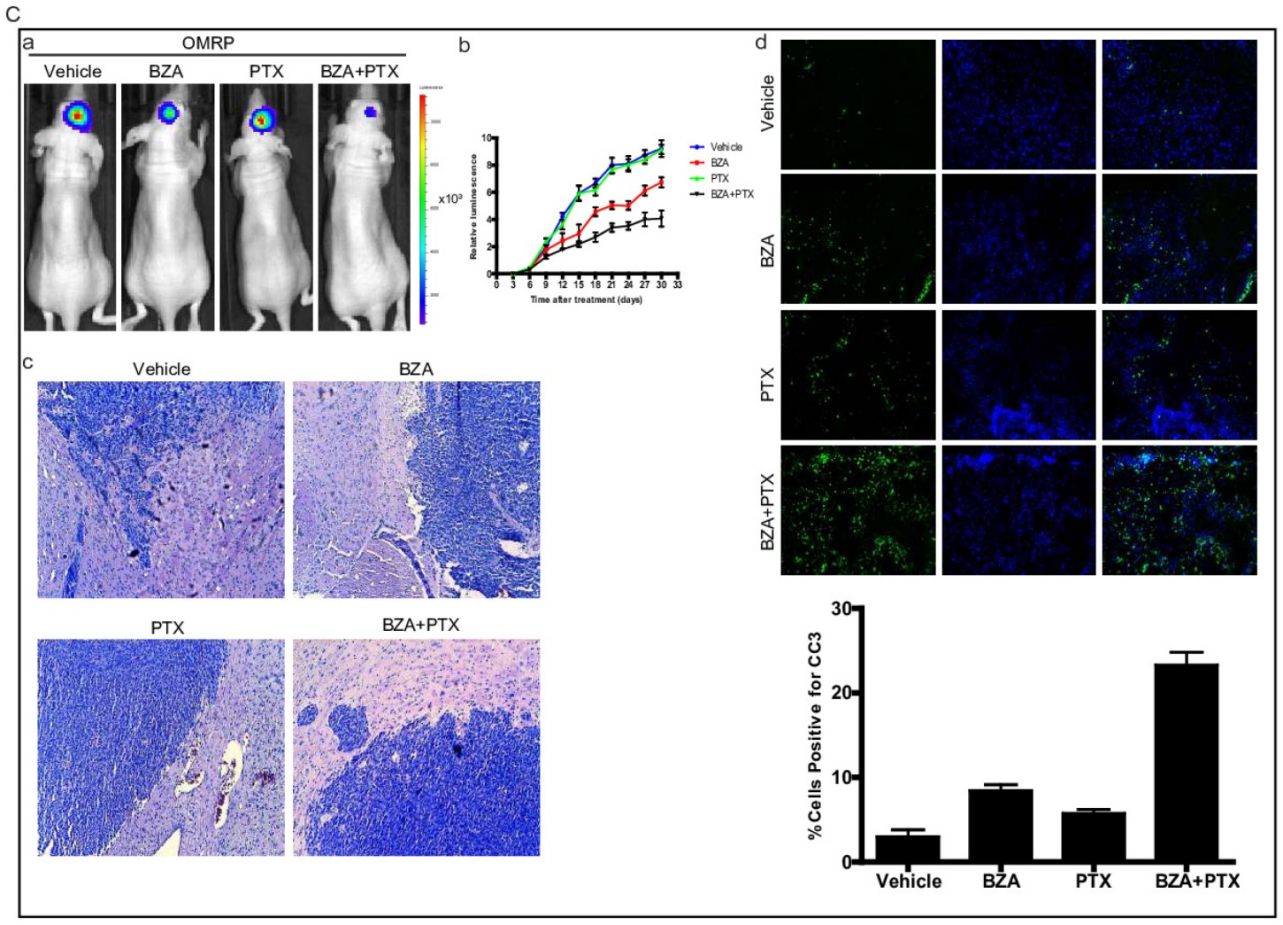
Bazedoxifene combined with paclitaxel further suppressed glioblastoma tumor growth in orthotopic GBM mouse model. **(A)**. Luciferase U251MG cells based orthotopic GBM mouse model showed that combination of bazedoxifene (BZA, 5 mg/kg) with paclitaxel (PTX, 10 mg/kg) remarkably suppressed tumor growth. **(a)**. Representative IVIS images. **(b)**. Tumor growth rate measured by IVIS.** (c)**. HE staining.** (d)**. Western blotting detection of EMT related markers and YAP signaling. **(e)**. immunofluorescence staining of cleaved caspase 3. **(B)**. Luciferase D456 cells based orthotopic GBM mouse model further confirmed that combination of bazedoxifene with paclitaxel remarkably suppressed tumor growth. **(a)**. Representative IVIS images.** (b)**. Tumor growth rate measured by IVIS. **(c)**. HE staining. **(d)**. Western blotting detection of EMT related markers and YAP signaling. (e). immunofluorescence staining of cleaved caspase 3. **(C)**. Luciferase OMRP cells based orthotopic GBM mouse model further confirmed that combination of bazedoxifene with paclitaxel remarkably suppressed tumor growth. **(a)**. Representative IVIS images. **(b)**. Tumor growth rate measured by IVIS.** (c)**. HE staining. **(d)**. immunofluorescence staining of cleaved caspase 3. *p<0.1; **p<0.01; ***p<0.001

To translate our in vitro findings into pre-clinical animal model, we sought to test the therapeutic efficacy of bazedoxifene either alone or in combination with paclitaxel in orthotopic GBM mouse model. First, we directly implanted 1X10^6^ luciferase U251MG cells into the brain of nude mice. After tumor reached to appropriate size, we randomly divided them into four groups and orally gave the following therapies every other day: 1) vehicle; 2) bazedoxifene (5 mg/kg); 3) paclitaxel (10 mg/kg); 4) bazedoxifene plus paclitaxel. Tumor growth rate was measured by IVIS. 4 weeks later, mice were sacrificed. Results showed that tumor growth (**Figure 6A, a-b**) was dramatically suppressed in bazedoxifene/paclitaxel treated group, which was also confirmed by HE staining (**Figure 6A, c**). Moreover, the alterations of EMT markers and YAP signaling were consistent to our in vitro results (**Figure 6A, d**). We also observed an enhanced levels of cleaved caspase 3, measured by immunofluorescence staining of frozen tissues, in the combinational treatment. We also confirmed these findings in D456 and OMRP based orthotopic GMB model. Results consistently showed that bazedoxifene could enhance paclitaxel efficacy to suppress glioblastoma tumor growth (**Figure 6B-C**).

## Discussion

Glioblastoma is the most common and lethal brain tumor in adults 22. It can be classified as primary or secondary malignant brain tumor dependent of cell origination 22. Primary tumor is the most common form of glioblastoma and is more lethal to people. While, secondary glioblastoma originates from a lower-grade astrocytic tumor and may progress into high-grade tumor over time. Even although current treatments such as surgery, radiation and chemotherapy improve patients' lives a lot 13, 15, the poor survival rate puts finding novel therapeutic strategies into the first place. Here, we first found that bazedoxifene could suppress cell growth and cell invasion of glioblastoma. Additionally, bazedoxifene combined with paclitaxel had better efficacy to inhibit glioblastoma progression. Our study finds a novel therapeutic option for the treatment of glioblastoma.

The incidence of glioblastoma has a gender difference: men are approximately 1.5-2 times more likely to have glioblastoma than women 11. In addition, previous study had demonstrated that glioblastoma cells were easily to develop tumor in male mice than in female mice 9. However, another study pointed that estrogen could promote glioblastoma tumor growth in orthotopic mouse model 1. These inconsistent results may tell us that ERα and ERβ play different roles in glioblastoma carcinogenesis and development. As the third generation selective estrogen receptor modulator, bazedoxifene can act as either estrogen agonist or estrogen antagonist dependent of cancer type. In this study, we found that bazedoxifene treatment evidently reduced the expression of estrogen receptor alpha (ERα), implying ERα plays tumor promoting role in glioblastoma development. More efforts should be made to dissect the underlying mechanisms by which ERα promotes glioblastoma progression.

The inhibitory effect of bazedoxifene on IL-6/GP130 signaling has been approved in several cancers including triple negative breast cancer, pancreatic cancer and head/neck cancer 5, 19, 23. Previous works showed that bazedoxifene bonded to GP130 D1 domain through the hot spots Ile83, Phe36, Tyr94, and Asn92, which blocked the entry of IL-6 to GP130 and leaded to the inhibition of downstream signaling such as AKT, STAT3 and ERK 19. As significant role of IL-6/GP130 in glioblastoma has been recognized long time ago. Glioblastoma samples tended to express higher levels of IL-6 compared to those in healthy brains 4. And IL-6 was a poor prognostic factor for glioblastoma patients 17. Moreover, GP130 as well as its downstream STAT3 signaling was tightly associated with the stemness of glioblastoma stem like cells 20: GP130 blocking antibody drastically stem-like properties in GSCs. All these data indicate that IL-6/GP130 signaling plays essential role in the initiation and progression glioblastoma and targeting this pathway may overcome glioblastoma development. As IL-6/GP130 inhibitor, bazedoxifene opens a new window for glioblastoma treatment.

The oncogenic role of YAP in Hippo pathway has been approved in glioma by many groups. YAP overexpression promoted glioma cell growth by altering Wnt/beta-catenin 21. Another study also demonstrated that YAP promoted cell migration and invasion by up-regulating N-cadherin and twist 27. Consistently, our data also displayed that bazedoxifene treatment accelerated YAP phosphorylation. However, the underlying mechanism by which bazedoxifene enhances YAP phosphorylation and leads its degradation is still unclear for us. We hypothesize that there is cross-talk between IL-6/GP130 and YAP. Therefore, deactivation of GP130 by bazedoxifene may accelerate YAP phosphorylation and degradation.

In summary, our data defines the anticancer activity of bazedoxifene either alone or in combination with paclitaxel in glioblastoma in vitro and *in vivo* and builds strong rational to develop bazedoxifene as another therapeutic drug for glioblastoma patients.
